# Developmental bias as a cause and consequence of adaptive radiation and divergence

**DOI:** 10.3389/fcell.2024.1453566

**Published:** 2024-10-16

**Authors:** Corin Stansfield, Kevin J. Parsons

**Affiliations:** School of Biodiversity, One Health & Veterinary Medicine, University of Glasgow, Glasgow, United Kingdom

**Keywords:** phenotypic plasticity, plasticity-led evolution, parallel evolution, evolvability, eco-evo-devo, extended evolutionary synthesis, ecotype evolution, quantitative genetics

## Abstract

Efforts to reconcile development and evolution have demonstrated that development is biased, with phenotypic variation being more readily produced in certain directions. However, how this “developmental bias” can influence micro- and macroevolution is poorly understood. In this review, we demonstrate that defining features of adaptive radiations suggest a role for developmental bias in driving adaptive divergence. These features are i) common ancestry of developmental systems; ii) rapid evolution along evolutionary “lines of least resistance;” iii) the subsequent repeated and parallel evolution of ecotypes; and iv) evolutionary change “led” by biased phenotypic plasticity upon exposure to novel environments. Drawing on empirical and theoretical data, we highlight the reciprocal relationship between development and selection as a key driver of evolutionary change, with development biasing what variation is exposed to selection, and selection acting to mold these biases to align with the adaptive landscape. Our central thesis is that developmental biases are both the causes and consequences of adaptive radiation and divergence. We argue throughout that incorporating development and developmental bias into our thinking can help to explain the exaggerated rate and scale of evolutionary processes that characterize adaptive radiations, and that this can be best achieved by using an eco-evo-devo framework incorporating evolutionary biology, development, and ecology. Such a research program would demonstrate that development is not merely a force that imposes constraints on evolution, but rather directs and is directed by evolutionary forces. We round out this review by highlighting key gaps in our understanding and suggest further research programs that can help to resolve these issues.

## 1 Introduction

The neo-Darwinian view of evolution commonly proposes that random genetic mutations lead to random phenotypic variation, which is then sorted by natural selection (Gregory, 2009). Through this view selection and genetic allele frequency changes have been at the centre of evolutionary enquiry over the past decades, from the emergence of evolutionary ecology (Endler, 1986) toward the incorporation of genetics (Mousseau et al., 2000), and ultimately genomic data. However, decades of molecular, developmental, and theoretical findings have shown that this view is limited and understates the role of development in driving evolutionary change (Laland et al., 2015; Muller, 2007). Phenotypic variation is the target of selection and arises through a range of developmental processes. Far from providing a blank slate for selection to act on, it is now understood that phenotypic development is “biased,” in that developmental systems respond to genetic and environmental perturbations in non-random ways (Uller et al., 2018). Indeed, it has been demonstrated that both phenotypic patterns of mutational variation (Braendle et al., 2010; Houle et al., 2017) and trajectories in multivariate phenotypic space are biased in nature (McGlothlin et al., 2018; Rohner et al., 2022; Schluter, 1996). This suggests that developmental processes impose structure upon phenotypic variation, including biases that may or may not align with patterns of macroevolutionary divergence (Houle et al., 2017; McGlothlin et al., 2018; Rhoda et al., 2023). However, how these biases shape, and are shaped, by evolutionary change is poorly understood, and requires a synthesis of ideas about how adaptation unfolds, and how phenotypic variation arises.

Investigating adaptive radiations may facilitate such a synthesis, as they can address both generative developmental processes and the sorting processes of selection. The predominant view in adaptive radiation research – ecological speciation – has focused more upon the contribution of selection. Adaptive radiations are characterised by the explosive diversification and speciation within a lineage, often triggered by the colonisation of a novel environment (Schluter, 2000). Such radiations have produced much of earth’s diversity (Wilson, 1999), including some of the most well-characterised study systems in evolutionary biology, such as African Rift Lake cichlids (Santos and Salzburger, 2012), Galapagos finches (Grant and Grant, 2002), and Caribbean anoles (Losos, 2011). However, whilst a growing body of work has investigated the role of development in adaptive radiations (e.g., Abzhanov et al., 2004; Maan and Sefc, 2013), the links between developmental bias and adaptive radiations have been neglected. This is despite many of the defining features of adaptive radiations suggesting a role for developmental bias. Therefore, we contend that radiating lineages could be used to reconcile developmental bias with adaptive evolution.

In this review, we will argue that many classic characteristics of adaptive radiations imply that developmental biases are both causes and consequences of these evolutionary patterns (Table 1). Specifically, these classic features are i) common ancestry within a radiating lineage; ii) rapid evolution along evolutionary “lines of least resistance,” often manifesting in parallel evolution; iii) the subsequent repeated and parallel evolution of ecotypes; and more recently the idea that iv) evolutionary change is “led” by biased phenotypic plasticity upon exposure to novel environments (Levis and Pfennig, 2019). In the process, we hope to demonstrate that adaptive radiations provide unprecedented opportunities to study developmental biases, and to integrate adaptive evolution and development. Finally, we suggest areas of research that will be required to fully appreciate the role that developmental biases play in adaptive radiations and divergence.

**TABLE 1:** Properties of adaptive radiations that can be better understood by integrating developmental bias into our thinking.

Property of adaptive radiations	Conventional perspective (neglecting development)	Incorporating developmental bias
Rapid evolution	Adaptive evolution occurs through random mutations that are unguided in their phenotypic consequences. Additional role for relaxed selection in a novel environment	Phenotypic consequences of mutations and environmental inputs are non-random (biased), and evolution can be accelerated if such biases align with axes favored by selection. Biases can evolve, and this may be facilitated by the release of competition
Parallel evolution	Similar selection regimes lead to similar phenotypic outcomes	In order to evolve in parallel, lineages must have similar patterns of bias, or be able to diverge along similar trajectories. These biases can evolve. If a lineage encounters an environment an ancestor has already adapted to, evolution may be accelerated if “current” biases reflect past selection
Radiating lineages have a “competitive advantage”	Certain lineages radiate because they claim a competitive advantage. Often, such successful colonisers are described as “generalists”	Lineages that gain a competitive advantage may do so if biases align with selection or evolve to do so before competitors. “Generalists” may show flexibility in these biases
Facilitated by ecological release	Adaptive radiations begin when a lineage invades a new environment. This “ecological release” from competition allows diversification into previously uninhabited niches	Shifts in developmental biases may break constraints. This “developmental release” would allow invasion into novel regions of morphospace. Thus, adaptive divergence and radiation may not require environmental changes
Repeated emergence of “ecotypes”	Radiations are characterised by the repeated emergence of “ecotypes” (or “ecomorphs”), characterised by the co-occurrence of several ecologically-relevant traits. Ecotypes emerge due to selection on this “complex” of traits	Ecotype emergence is driven by patterns of covariation that link such traits, and thus represents different points on a “line of least resistance.” Once these patterns of covariation evolve once within a lineage, future divergence along this axis will be facilitated and accelerated
Radiating lineages are characterised and facilitated by common ancestry	Genetic similarity will increase the likelihood of parallel evolution	Common ancestry may correspond to more similar patterns of developmental bias, thus increasing the likelihood of parallel evolution
Adaptive divergence is often seeded through phenotypic plasticity	Plasticity-led evolution can accelerate adaptation and ensure phenotype-environment correlations	Responses to environmental and genetic perturbations are similarly biased by a shared developmental system. Closely-related lineages may show similarly-biased plastic responses, driving parallelism. Plasticity-led evolution alters the environment-phenotype and genotype-phenotype maps, increasing the likelihood of similar plastic responses in the future

## 2 A primer on developmental bias

Darwin was the first to observe and recognise both the discontinuities of variation and the importance of understanding its underlying mechanisms, but lacking knowledge of genetics and development he was unable to provide such explanations (Darwin, 1859; Gliboff, 2023). Since his time, a coherent understanding of genetics preceded knowledge of development, leading to a gene-centric view of evolution referred to as the “modern synthesis,” which remains central today (Laland et al., 2014a). In this highly quantitative view of evolution, any potentially biases in the distribution of mutational effects are overpowered by selection (Fisher, 1930; Yampolsky and Stoltzfus, 2001), thus relegating development to the role of an uninteresting and undirected process that produces the substrate for selection to “sculpt” evolution to its liking (Hendrikse et al., 2007). Findings from molecular biology, genetics and paleontology led to a renewed interest in development at the tail end of the 20th century, led by researchers such as Stephen Jay Gould (Gould, 1977; Gould, 1989; Gould and Lewontin, 1979; Alberch 1980; Alberch 1989), and John Maynard-Smith (Maynard-Smith et al., 1985) amongst others. This research program sought to understand “rules of development,” which were typically viewed as constraining forces that influenced evolution only by impeding selection (Gould, 1989), with Maynard-Smith et al. (1985) recommending “extreme caution in claiming that such constraints are responsible for evolutionary trends.” It was not until the turn of the millennium when researchers began to understand these developmental “rules” as forces that could accelerate or improve the efficacy of evolution (Arthur, 2001; Schluter, 1996). Thus, the need to integrate evolution and development, and “extend” the modern synthesis (Pigliucci, 2007), was recognized.

The “extended evolutionary synthesis” (EES) focuses on bridging the explanatory gap between genotype and phenotype, by understanding how developmental processes can explain evolutionary trends (Laland et al., 2015; Pigliucci, 2007). In this framework, embryos are not simply a collection of genes, but are developmental systems capable of guiding their own ontogenetic trajectory (Laland et al., 2014b; Walsh, 2015). Furthermore, evidence from models of tooth development (Kavanagh et al., 2007) and pattern formation (Turing, 1952; Yamaguchi et al., 2007) demonstrate that adaptive variation can be produced by interactions between morphogenic elements, often in dynamic and reciprocal fashion, that suggest levels of developmental causation occurring above the level of genes (Müller and Newman, 2003). The overarching conclusion of this research program so far is that evolvability - the ability to produce adaptive and heritable phenotypic variation (Kirschner and Gerhart, 1998) - is itself capable of evolving (Pigliucci, 2008; Wagner and Altenberg, 1996). In other words, developmental architectures evolve to become “biased” towards adaptive regions of phenotypic space (Uller et al., 2018).

## 3 Characteristics of adaptive radiations that imply role for biases

### 3.1 Common ancestry

The fact that members of an adaptive radiation necessarily share a recent common ancestor (Schluter, 2000) allows us to study how the combined effects of selection and development determine the generation and persistence of phenotypic variation within a radiating lineage. Note that adaptive radiations do not simply reflect the sorting of ancestral variation into new forms, but rather are characterised by the production of novel and exaggerated variants that far exceeds that observed in the ancestor. However, common ancestry means that members of a radiation share a common ancestral developmental system making it more likely that the responses of lineages to various environmental and genetic inputs are biased along similar developmental trajectories (Parsons et al., 2020; Schluter, 1996) (Figure 2A). This ultimately ties to the themes we discuss next.

### 3.2 Rapid evolution along lines of least resistance

**FIGURE 1:**
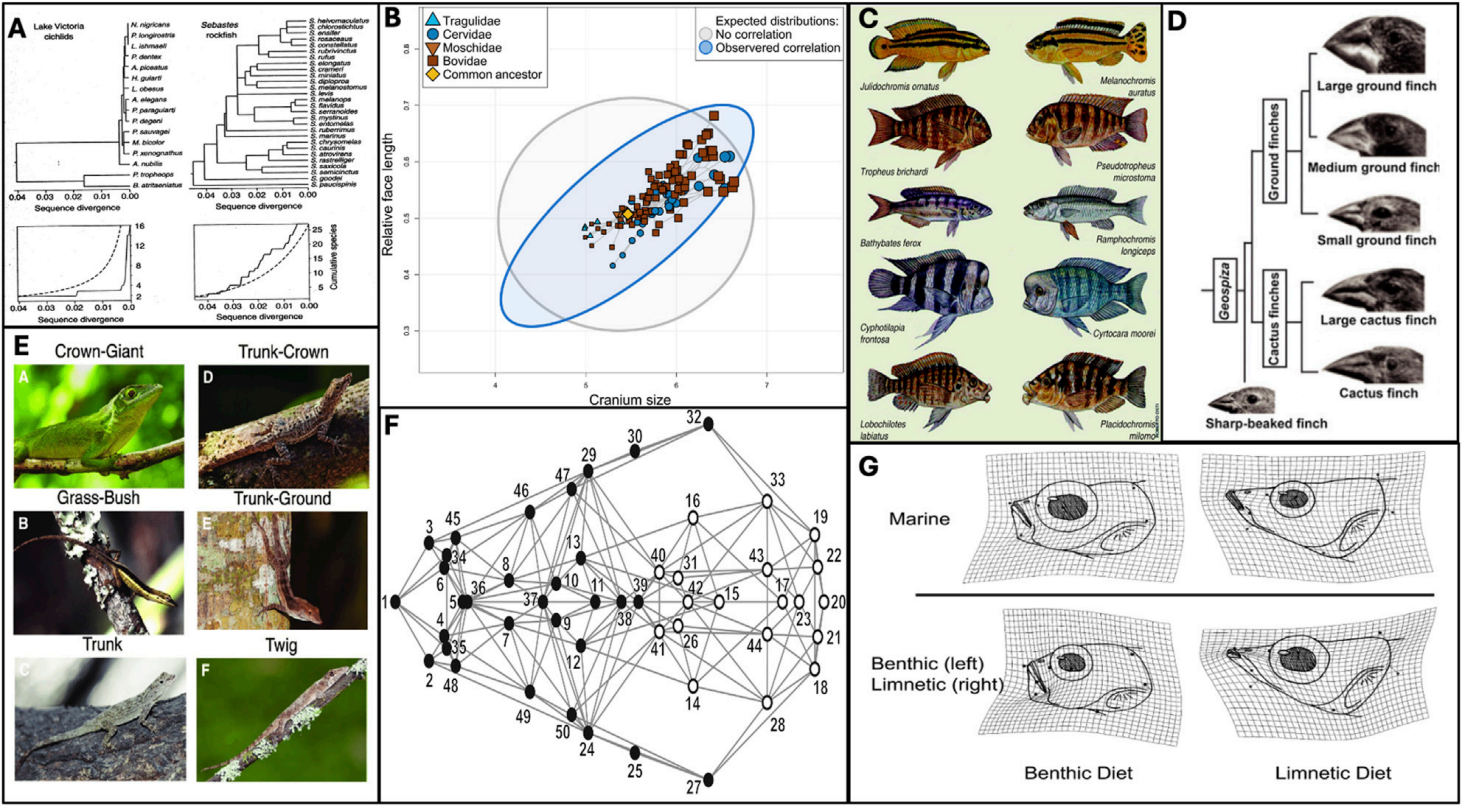
Defining features of adaptive radiations suggest a role for developmental bias. **(A)** If lines of least resistance align with axes of selection, evolution can be greatly accelerated. Cichlid speciation (left) and sequence divergence is rapid, surpassing neutral expectations, and evolutionary rates in a non-radiating family, *Sebastes* rockfish. Figure from Schluter (2000) p.15. **(B)** Evolution tends to follow “lines of least resistance.” Rhoda et al. (2023) found that ruminant cranial evolution follows a line of least resistance driven by an allometric relationship between face length and cranium size. Thus, variation is restricted to a narrower region of phenotypic space (blue) than would be expected under the null hypothesis of isotropic variation (grey). **(C)** Similar patterns of developmental bias within a lineage can contribute to parallel evolution. Many similar species have emerged independently in three African Rift Lakes - Malawi, Victoria and Tanganyika. Diagram from Stiassny and Meyer (1999) shows fish from lake Tanganyika on the left, and similar varieties from Lake Malawi on the right. **(D)** Adaptive radiation is characterised by ecotypic divergence, driven by altered patterns of covariation. Galapagos finches represent a textbook example, due to divergence in skull and beak morphology in response to different feeding niches. Figure from Mallarino et al. (2011). **(E)** The *Anolis* adaptive radiation is characterised by the repeated emergence of six ecotypes, inhabiting different microhabitats. Figure from Huie et al. (2021) **(F)** Adaptive radiation are often facilitated by “key innovations,” seeded through altered patterns of covariation that bias evolution towards certain regions of phenotypic space. Drake and Klingenberg (2010) demonstrated that the canine skull is composed of two modules, mandibular (black) and cranial (white), and associated this developmental lability with the diversity observed within domestic dog breeds, which eclipses that of the entire carnivora family. **(G)** Adaptive divergence is often seeded through phenotypic plasticity, which is biased in nature. Wund et al. (2008) demonstrated that specialised benthic and limnetic feeding morphologies (bottom), characterised by patterns of covariaton, could be recapitulated plastically in the marine ancestor (top). Such “plasticity-led evolution” is a characteristic feature of adaptive radiations.

**FIGURE 2:**
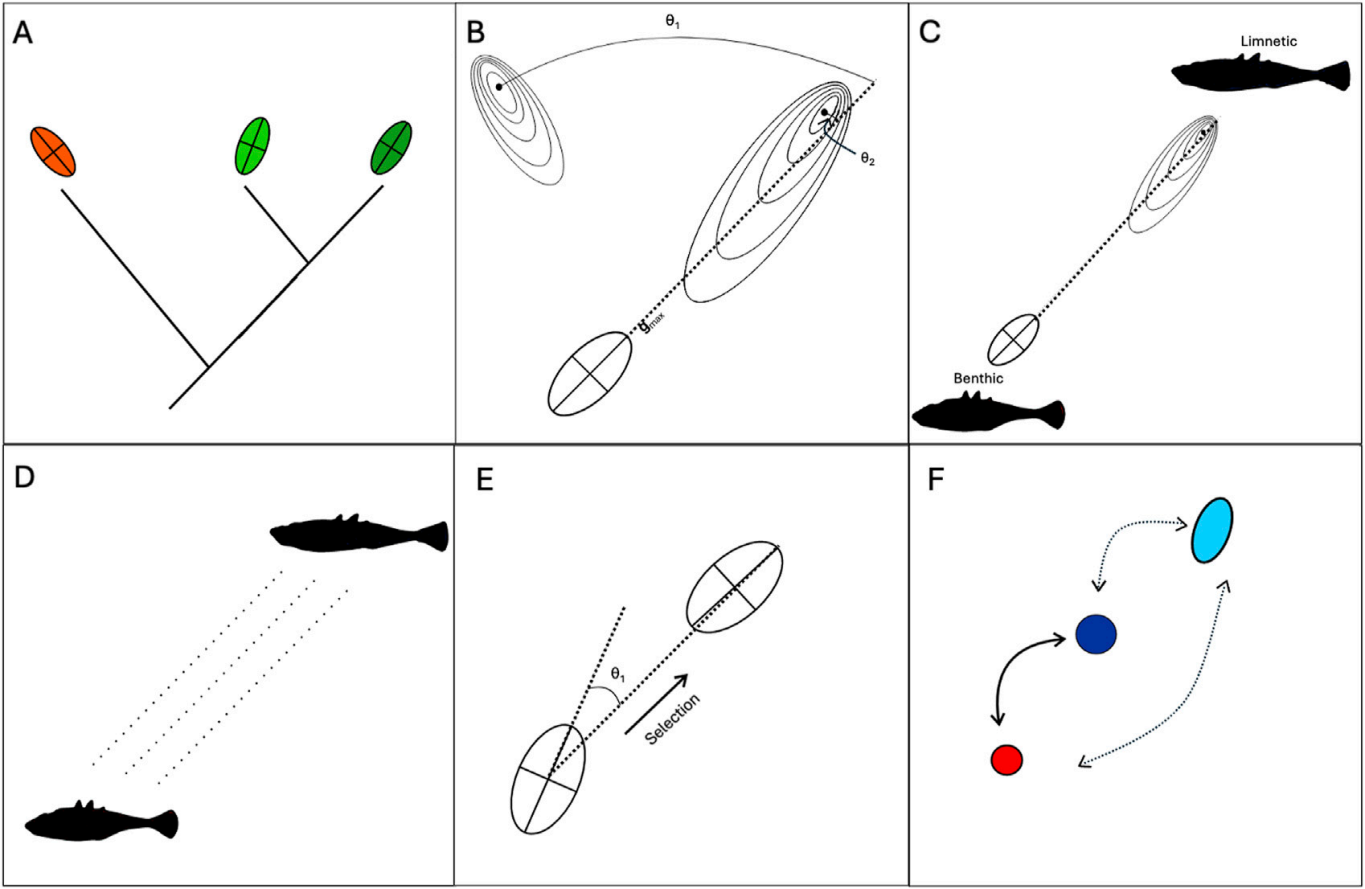
Bias as a cause and consequence of adaptive radiations and divergence. **(A)** Common ancestry may lead to similarly-biased developmental systems. As a result, parallel evolution may be more likely within a lineage, or one lineages may be able to evolve in the face of ecological “opportunity” while another lineage cannot. **(B)** Evolution tends to occur along “lines of least resistance,” captured by G_max_ – the axis along which the most additive genetic variation is produced. If G_max_ aligns with the fitness landscape (θ is small), then adaptive evolution may by accelerated (e.g., θ_1_). Conversely, if G_max_ for a population or lineage is not aligned with the fitness landscape (θ is large, e.g., θ_2_) adaptation may be slowed or constrained entirely. Hence, biases can be viewed as permissive and constraining forces. **(C)** Lines of least resistance typically correlate with multiple ecotypes. For example, Schluter’s (1996) line of least resistance for threespine stickleback contained slender, shallow-bodies limnetic forms at one end and deep-bodied limnetic forms at the other end. **(D)** Parallel evolution of ecotypes can occur through a combination of parallel selection pressures and parallel patterns of bias. Benthic-limnetic divergence has occurred numerous times independently in sticklebacks, as has the emergence of similar divergent phenotypes in response to different environmental gradients. **(E)** Adaptive divergence results in altered patterns of developmental bias. Thus, biases can be viewed as consequences, as well as causes, of adaptive divergence and radiation. Future evolution can be accelerated if it occurs along axes previously favoured by selection. **(F)** Rapid evolution can occur via biased phenotypic plasticity. “Learning” can occur in gene regulatory networks, allowing rapid plastic switching between ecotypic forms that can become refined over time (hard arrows). Developmental noise induced by novel environmental cues will further be directed along axes previously favoured by selection (dashed arrows).

Another defining feature of adaptive radiations are their greatly accelerated rates of diversification and speciation (Gillespie et al., 2020; Schluter, 2000). As a famous example, over 500 species of cichlids have emerged within Lake Victoria from common ancestry in approximately 10,000 years (Galis and Metz, 1998), involving accelerated rates of speciation (Henning and Meyer, 2014) and the emergence of diverse morphologies (Witte et al., 2008), colour patterns (Maan and Sefc, 2013), and life-histories (Ribbink, 1990) (Figure 1A). In search of a general mechanism for such rapid rates of evolution, Schluter (1996) used a quantitative genetics approach to demonstrate that phenotypic evolution in threespine stickleback was in closer alignment to **g**_max_ - the multivariate direction of greatest additive genetic variance within a population – than would be expected by chance. This phenomenon has since been confirmed by a large body of literature (Bégin and Roff, 2004; Blows and Higgie, 2003; Brattström et al., 2020; Marroig and Cheverud, 2005; McGlothlin et al., 2018; McGuigan et al., 2005; Rhoda et al., 2023; Figure 1B; Renaud et al., 2006; Rohner and Berger, 2023; Walter et al., 2018), although counter-examples also exist (Badyaev and Hill, 2000; Merilä and Björklund, 1999). Quantitative-genetic models (Lande, 1979) then suggest that evolutionary trajectories do not necessarily follow the direction of fastest ascent in the fitness landscape (referred to as the “selection gradient”), but rather are biased towards **g**_max,_ which Schluter calls the “genetic line of least resistance.” While misalignment between selective and developmental axes can constrain adaptive evolution and reduce fitness gains, strong alignment can accelerate it (Figure 2B). For example, Marroig and Cheverud (2005) showed that evolutionary rates decreased by a factor of 3–4 when selection and **g**_max_ (i.e., development) are not aligned. However, while evidence suggests that evolution tends to occur along lines of least resistance initially, without understanding the stability of the structure of multivariate phenotypic variation over generations the predictive power of such models is limited.

Indeed, while the quantitative genetics program has yielded many important evolutionary insights, the **G**-matrix – a matrix of trait variances and covariances from which **g**_max_ is derived – carries predictive power only from one generation to the next (Pigliucci, 2006), as it is itself subject to drift and selection. Thus, how such a research program can bridge micro- and macroevolution is a persistent issue (Arnold et al., 2001). For example, Boell (2013) found that the **G**-matrix was unique for each generation, while Björklund et al. (2013) and Doroszuk et al. (2008) found fluctuations in the **G**-matrix over relatively short time frames. Schluter (1996), based on the models of Lande (1979) and Via and Lande (1985), predicted that his model carried predictive power only for the initial stages of divergence, and that selection should overpower developmental constraints given enough time. Nevertheless, (Schluter, 1996) showed that stickleback evolution had followed the line of least resistance for at least 4 million years, as far back as the data allowed inference. Similarly, (Renaud et al., 2006) observed that evolution in rodents aligned with **g**_max_ for 6.5 million years, before eventually diverging, and evidence from *Anolis* (McGlothlin et al., 2018), *Sepsidae* flies (Rohner and Berger, 2023), *Drosophila* (Houle et al., 2017), and *Onthophagus* dung beetles (Rohner et al., 2022) show alignment between lines of least resistance and macroevolutionary patterns seemingly persisting over tens of millions of years. Thus, in spite of the ephemeral nature of the **G**-matrix, a meta-analysis by Arnold et al. (2008) found that several measures, and particularly the orientation of **g**_max_, were broadly similar in 74% of studies comparing experimental treatments, sexes, populations, and species. These results suggest that **g**_max_, and thus the underlying developmental processes – may play a role in dictating macroevolutionary patterns.

While Houle et al. (2017) argue that the alignment between lines of least resistance and macroevolution is suggestive of evolutionary constraint, it could also be argued that the **G**-matrix and **g**_max_ are subject to selection and are therefore capable of evolving. Theoretical models suggest that pleiotropy (Jones et al., 2003; Jones et al., 2007) and epistasis (Jones et al., 2014)–the processes determining patterns of covariation – can be molded by selection so that the distribution of mutations becomes aligned with the fitness landscape. The explanation that the **G**-matrix and lines of least resistance are evolvable is preferred by Rohner and Berger (2023), McGlothlin et al. (2018), and Schluter (1996). Indeed, Schluter (1996) remarks that the **G**-matrix provides explanatory power for patterns of variation that are demonstrably the result of natural selection, suggesting alignment between selective and development explanations. To support this, the meta-analysis of Arnold et al. (2008) found that, although **G**-matrices and **g**_max_ were fairly stable across published studies, divergence was observed in species under divergent selection (Cano et al., 2004; Doroszuk et al., 2008; Eroukhmanoff and Svensson, 2011; Fong, 1989; Jernigan et al., 1994; Johansson et al., 2011; Roff et al., 2004; Shirk and Hamrick, 2014; Land et al., 1999), further suggesting malleability of these matrices. Direct evidence comes from Walter et al. (2018), who found **G**-matrix divergence between wildflower ecotypes evolving in different microhabitats, suggesting developmental divergence driven by diverging selection regimes. When taken together, these data suggest that **G**-matrices, and thus the underlying developmental processes, impose structure on the phenotypic variation made available to selection, and these biases evolve and subsequently influence macroevolution. Understanding both the stability and evolvability of these biases is required to fully understand their role in adaptive radiations.

A persistent criticism of the modern synthesis is that it fails to consider developmental processes, and thus can explain what happens to phenotypic variation that is generated but does not provide insights into how this variation originates. A similar criticism can be made of quantitative genetics, which typically uses modelling approaches that are naïve to underlying development processes (Hansen and Houle, 2008). However, a growing number of interdisciplinary researchers are now successfully integrating development and quantitative genetics to help bridge the gap between micro- and macroevolution and improve the predictive powers of such models. Recently, Mongle et al. (2022) and Machado et al. (2023) both utilised a study system with well understood developmental “rules”– the inhibitory cascade model (ICM) that describes mammalian molar development – to study evolution within a “biologically-informed” morphospace. Predictable interactions between inhibitory and activating factors maintain a consistent ratio of molar sizes and create a discontinuous morphospace of patterns that can be produced by development, thus limiting the trajectories that evolution can take. Indeed, such a morphospace was able to unite micro- and macroevolutionary patterns, leading Machado et al. (2023) to suggest that observed variation is the result of stabilising selection that is “enforced” by development. Thus, the existing literature suggests that evolution tends to occur along axes with high additive genetic variation, but integration of these quantitative genetic metrics with developmental knowledge will further improve their predictive power.

### 3.3 Parallel and repeated evolution of ecotypes

The lines of least resistance as described by Schluter (1996) often describe axes of ecotypic divergence. “Ecotypes” represent body plans defined by a set of ecologically relevant traits that tend to covary together. Radiating lineages are thought to evolve distinct ecotypes in response to different environments that are encountered during colonisation (Schluter, 2000). Theory suggests that correlated selection acting on a set of traits and their respective covariances should stabilize the **G**-matrix so that the line of least resistance aligns with a ridge on the fitness landscape (Jones et al., 2004). This ridge may straddle two or more fitness peaks that represent ecotypes, thus allowing accelerated transitions between body plans sharing patterns of trait covariance that have previously been favoured by selection. For example, (Schluter, 1996) line of least resistance for sticklebacks had “limnetic” morphologies at one end, possessing smaller and more slender bodies than “benthic” counterparts at the other end of the axis (Figure 2C). Similarly, Caribbean anoles have repeatedly evolved into a limited set of ecotypes, each of which corresponds to a different microhabitat (Huie et al., 2021; Losos, 2011). These ecotypes are primarily differentiated by size and limb proportions, which corresponds to the line of least resistance observed by McGlothlin et al. (2018). Freedom to vary along ecologically relevant axes of covariation can facilitate rapid evolution. For example, the capacity to rapidly produce well-adapted benthic and limnetic trophic morphologies has been strongly linked to the prodigious evolution displayed by cichlids (Albertson and Kocher, 2006). The propensity for the above groups to recapitulate these ecotypic forms through plasticity in lab settings (Calsbeek et al., 2007; Parsons et al., 2016) further suggests a prodigious capacity to vary along these axes in the face of genetic or environmental inputs into development. In all, ecotypic divergence driven by developmental lability along an ecologically determined set of trait covariations is a prominent feature of adaptive radiations and may contribute to accelerated rates of diversification.

Ecotypic divergence, and subsequent adaptive radiations, have been observed on numerous occasions to be driven by “key innovations” – such as varied beak morphologies in Galapagos finches (Grant and Grant, 2002) – that typically involve altered patterns of covariation between traits. Modularity – the semi-independence of traits and/or developmental programs (Schlosser and Wagner, 2004) – has been shown to be a driver of numerous adaptive radiation events by providing the “degrees of freedom” required to explore morphospace. For example, the developmental decoupling of the oral and pharyngeal jaws in cichlids has long been linked to trophic diversification by allowing these semi-independent structures to evolve distinct functions (Liem, 1973; Parsons et al., 2012 - but see Conith and Albertson, 2021). Similarly, division of the canine skull into cranial and mandibular modules has been implicated as a factor in the diversification of dogs under domestication, the diversity of which eclipses the entire *carnivora* family (Figure 1F) (Drake and Klingenberg, 2010; Wilson et al., 2021). Modularity has been implied in the diversification of apes (Parins-Fukuchi, 2020) and carnivorous mammals (Law et al., 2022) amongst other radiating lineages, further implying a link between trait independence and evolutionary rates. Conversely, the coupling of traits - integration - has similarly been linked to rapid exploration of morphospace by allowing correlated trait complexes to evolve together, requiring minimal regulatory changes (Gerhart and Kirschner, 2007). For example, “domestication syndrome” describes rapid evolution due to the shared developmental origin of cell populations in neural crest cells (NCCs) (Wilkins et al., 2014). Artificial selection during domestication is predicted to lead to a reduced contribution of NCCs to developmental structures, leading to fewer cartilage, pigment and neuronal cells, and thus concomitant changes in linked traits (Lesch and Fitch, 2024; Trut, 1999; Wilkins, 2020). While the involvement of NCCs as an explanation for domestication syndrome has been controversial (see Gleeson and Wilson, 2023; Wright et al., 2020) evidence from “de-domestication” (Flyn, 2022) and the evolution of aggression (Feiner et al., 2024) suggest that the traits that contribute to domestication syndrome can easily covary in the other direction, demonstrating how integration at the developmental level allows rapid evolution along axes of covariation.

The repeated evolution of ecotypes at the level of species, assemblages, and taxa, further implicates a role for developmental biases (Figures 1C–E). For example, independent invasions of freshwater habitats by marine sticklebacks have led to the repeated parallel evolution along a marine-freshwater axis (Roberts Kingman et al., 2021) (Figure 2D). These marine and freshwater ecotypes bear anecdotal resemblance to benthic and limnetic ecotypes as described by Schluter (1996), as well as populations that have diverged along lake-stream (Ravinet et al., 2013), geothermal-ambient (Pilakouta et al., 2023), and mud-lava (Kristjánsson et al., 2002) axes, suggesting that shared patterns of covariation have led to adaptive divergence being channeled along the same or similar phenotypic axes. Benthic-limnetic divergence has been observed in a wide range of fish clades such as cichlids (Hulsey et al., 2013), labrids (Larouche et al., 2023), and several postglacial fishes (Parsons and Robinson, 2007; Skulason et al., 2019), suggestive of a taxa-level bias that has shaped, and perhaps been shaped by selection to align with this ecological axis, thus potentially accelerating evolution. This ecotypic divergence is well characterised in the African Rift Lake cichlid radiations, in which parallel patterns of trophic diversification have been identified (Cooper et al., 2010; Hulsey et al., 2013). These patterns have been shown to be underpinned by parallel patterns of craniofacial modularity (Parsons et al., 2012), suggesting an interactive role between selection and developmental bias in driving adaptive radiations.

However, whilst these replicated radiations present some of the most well-characterised evolutionary events, such patterns are observed less frequently than theory would suggest (Losos, 2010), with E.O. Wilson (1999) observing that “many clades fail to radiate although seemingly in the presence of ecological opportunity.” In these cases, the absence of an expected pattern is significant, and suggests that natural selection plays an important, but incomplete role as only a sorting mechanism in adaptive radiations. Explanations of parallel evolution must therefore look beyond parallel selection pressures and consider that the variation exposed to selection is produced by developmental systems that are biased and may be pre-disposed to producing variation in directions favoured by previous selection. Such biases may be particularly relevant when investigating populations that share common ancestry, as this may pre-dispose development along similar trajectories. A driving role for developmental processes is substantiated by the observation that parallel evolution is infrequently mirrored at the genetic level (Barghi et al., 2019; Conte et al., 2012; Poore et al., 2023), and can be achieved by mutating alternative genes in the same pathway (Steiner et al., 2009). Furthermore, replicated radiations tend to occur within the same clade (Losos, 2010), with repeated benthic-limnetic divergence across fish clades being an exception. Thus, phylogenetically related, but geographically distant, populations can follow similar evolutionary trajectories (e.g., threespine stickleback - Roberts Kingman et al., 2021), while other clades co-existing with radiating lineages fail to radiate themselves (e.g., Galapagos Mockingbirds – Arbogast et al., 2006; Losos, 2010). Hence, what have long been labelled as “contingencies” (Blount et al., 2018; Gould, 1977) or “phylogenetic constraint” (McKitrick, 1993) may reflect divergence in developmental systems, which may preclude or facilitate divergence along certain trajectories. For example, finches and honeycreepers have radiated on both the Galapagos islands and mainland South America while mockingbirds and thrushes, while present in both regions, have failed to radiate (Arbogast et al., 2006). Navalón et al. (2020) demonstrated links between clade divergence and altered patterns of integration between the beak and cranium, suggesting that these evolutionary patterns can be linked, at least in part, to intrinsic factors. Similarly, some butterfly taxa such as the *Mycalesina* subtribe have diversified into over 250 species originating from numerous radiation events spanning multiple continents (Brakefield, 2010; Kodandaramaiah et al., 2010), while most other butterfly clades are relatively species-poor. An alternative explanation is that radiating clades possess a competitive advantage, but this could also be interpreted as a developmental explanation, with niche expansion being facilitated by a labile developmental system. Looking at initial stages of colonisation, we should also ask if this evolvability was present in colonisers, or if it rapidly evolved in response to ecological opportunity. Thus, by understanding why some clades radiate while others fail to do so, we can understand how developmental factors co-exist and interact with other factors influencing evolutionary patterns.

### 3.4 Biased plasticity as a driver of adaptive divergence

Perhaps the defining quality of adaptive radiations is rapid evolution upon exposure to new environments. New environments should induce a plastic response, and there is now plentiful evidence that adaptive radiations are in some cases seeded by phenotypic plasticity – the capacity of a developmental system to produce phenotypic variation in response to environmental cues (Levis and Pfennig, 2019; West-Eberhard, 2003). Plasticity-induced trait variation can help populations persist in novel environments (“the Baldwin effect:” Baldwin, 1896; Simpson, 1953) and can then become refined (“accommodated”) and eventually “assimilated” whereby an environmental cue is no longer a requirement of its development (Waddington, 1953; West-Eberhard, 2003). Models of plasticity-led evolution, such as the flexible stem hypothesis (West-Eberhard, 2003), state that extant patterns of phenotypic diversity are partly determined by the plastic capacities of ancestral (“stem”) lineages (Parsons et al., 2016; Wund et al., 2008). The capacity of ancestral populations to produce derived ecotypes when reared in novel environments (Parsons et al., 2016; Rohner et al., 2022; Wund et al., 2008; Figure 1G) further suggests a role for plasticity-led evolution in adaptive radiations (Levis and Pfennig, 2019; Pfennig et al., 2010).

Although plastic responses to novel environments have long been considered as a “noisy” variable in evolution (Murren et al., 2015), increasing evidence suggests that plastic “noise” is indeed biased. West-Eberhard (2003) proposed that genetic and environmental cues can be viewed interchangeably, by both providing sources of information into the same developmental system. Indeed, a recent meta-analysis by Noble et al. (2019) demonstrated that noise induced by genetic and environmental perturbations was biased along the same axis, while Rohner et al. (2022) found that genetic and environmental interventions biased *Onthophagus* beetle horn development in similar ways. The production of phenocopies – phenotypic forms that can be replicated in the lab through non-genetic manipulations – suggests that genetic and environmental inputs are processed by the same developmental system, with its inherent biases (Alberch and Gale, 1985; Parsons et al., 2014; Wund et al., 2008). If closely related species show similar patterns of developmental bias, we may expect similar plastic responses upon exposure to the same environmental cue (s). Under such a scenario, plasticity-led evolution would impose a phylogenetic signal due to developmental biases, potentially explaining why replicated radiations tend to occur within lineages (Wilson, 1999). Thus, developmental biases may act to homogenise adaptive plastic responses in sister taxa, perhaps explaining patterns of benthic-limnetic divergence within and across fish clades (Larouche et al., 2023; Parsons and Robinson, 2007). The observation that ancestral plasticity tends to align with patterns of phenotypic variation (Radersma et al., 2020) further suggests a role for biased plasticity in determining the trajectories taken during adaptive divergence.

Understanding the role of developmental bias in driving phenotypic plasticity may ameliorate some of the oft-cited costs of plasticity. It is thought that plasticity must carry costs, otherwise organisms should be perfectly plastic and able to match any fitness optima (DeWitt et al., 1998), although empirical attempts to demonstrate such costs have rarely succeeded (Murren et al., 2015; Van Buskirk and Steiner, 2009). A commonly cited cost of plasticity is the risk of mismatches between organism and environment, or more broadly the production of phenotypes upon exposure to a novel environment that have not been “vetted” by selection (Murren et al., 2015). However, as we posit that plastic responses are produced by a biased developmental system, we would argue that plastic noise is far from random (e.g., Noble et al., 2019). Rather, we may expect that plastic responses will be closely aligned with past selection, thus falling closer to the phenotypes of parents and ancestors than would be expected by chance. If plasticity-led evolution is driven by extreme (“transgressive”) phenotypes, such leaps in morphospace may be aligned with such axes, producing “hopeful monsters” with underlying logic (Figure 2F) (Alberch, 1989). A view of plasticity that considers developmental trajectories rather than development of explicit phenotypes may also explain why plastic capabilities do not degrade over thousands or millions of years when the “machinery” associated with a trait lays dormant. Evolution may favour flexibility along a multivariate axis defined by a pattern of covariation, accelerating and refining plastic responses when a cue is re-encountered. Furthermore, a growing body of evidence suggests that the genetic architecture of traits is environment-dependent, with loci underpinning trait variation differing under experimental treatments (Bao et al., 2018; Conith et al., 2018; Ishikawa et al., 2017; Mathews et al., 2008; Messmer et al., 2009; Navon et al., 2021; Parsons et al., 2016; Snoek et al., 2017; Yadav et al., 2016; Zhang et al., 2010; Zogbaum et al., 2021). “Canalising” plasticity to produce discrete variants that have been refined by selection mitigates much of the stochasticity associated with plastic responses. In addition, modularisation of the environment-specific gene expression networks underlying plasticity can avoid disruptive pleiotropy and epistasis, thus, in the context of the specialist-generalist dichotomy pervasive in the plasticity literature, allow an organism to evolve specialisations for multiple sets of environmental conditions without encountering tradeoffs (Snell-Rood et al., 2010). For example, many *Mycalesina* butterfly species show polyphenisms, in which either a “wet season form” or a “dry season form” will develop in response to environmental conditions (Halali et al., 2024). These forms are primarily characterised by finely detailed (Prudic et al., 2015) and adaptive wing patterns (Brakefield and Frankino, 2009), but also show correlated changes in behaviour, physiology, and life-history. A developmental architecture that situationally shields developmental programs from selection may also relax selection, allowing the accumulation of cryptic genetic variation that enables the production of transgressive phenotypes (Snell-Rood et al., 2010). Thus, biased properties of development may ameliorate some of the costs and limitations attributed to phenotypic plasticity.

### 3.5 Bias as a cause and consequence of adaptive radiation

Central to the assertion that developmental bias is a cause and consequence of adaptive radiations are the observed mathematical equivalencies between the evolution of gene regulatory networks (GRNs) and learning in neural networks (Watson and Szathmáry, 2016). GRNs have been shown to demonstrate “Hebbian learning,” informally known as “fire together wire together” (Pavlicev et al., 2011; Watson and Szathmáry, 2016). Epistatic interactions between regulatory genes that confer a selective advantage will be strengthened while those that are deleterious will be weakened, leading the epistatic landscape to act as a logbook for prior selection. Hebbian learning allows GRNs to “generalise,” learning which interactions should and should not be conserved, and thus causing evolution to be guided to adaptive regions of morphospace containing both previously adaptive solutions and structurally similar novel phenotypes (Kouvaris et al., 2017; Parter et al., 2008). In extreme cases, adaptive solutions can be memorised and recalled if conditions demand. For example, (Rajakumar et al., 2012) demonstrated that several species of ant within the genus *Pheidole* were able to produce supersoldier castes when induced in the lab, suggesting this developmental program had been “memorised” for 45–60 million years despite not being used. Incorporating learning theory into evolution therefore means appreciating that developmental responses will be informed by past experience. Indeed, Brun-Usan et al. (2021) demonstrated that changes in the environment-phenotype map will lead to changes in the genotype-phenotype map, and vice-versa, thus aligning genetic and plastic responses along previously adaptive trajectories (Figure 2E). The utilisation of past information can also offer an explanation for the rapidity of evolution under adaptive radiations. Many of these radiations involve the repeated colonisation of similar habitats, for example, caused by fluctuating water levels in African Rift Lakes (Nevado et al., 2013), exposure to new freshwater habitats through deglaciation (Skúlason et al., 2019), or colonisation of emerging islands in an archipelago (Freed et al., 1987). Learning theory suggests that prior evolution along an environmental gradient could lead to flexibility along such axes in derived lineages. In such cases, radiating species may not need to mount an evolutionary response “from scratch,” instead riding the coattails of their ancestors. Work on regulatory circuits supports this theory, demonstrating that exposure to fluctuating selection, between environments that share structural regularities, led populations to regions of morphospace in which all solutions or adaptive “peaks” were readily accessible, in the best models through a single regulatory change (Kashtan and Alon, 2005; Parter et al., 2008). We therefore suggest that biased development can represent both causes and consequences of adaptive radiations by i) allowing “recall” of “memorised” adaptive solutions previously vetted by selection and ii) by channelling noise along previously fruitful axes.

**FIGURE 3:**
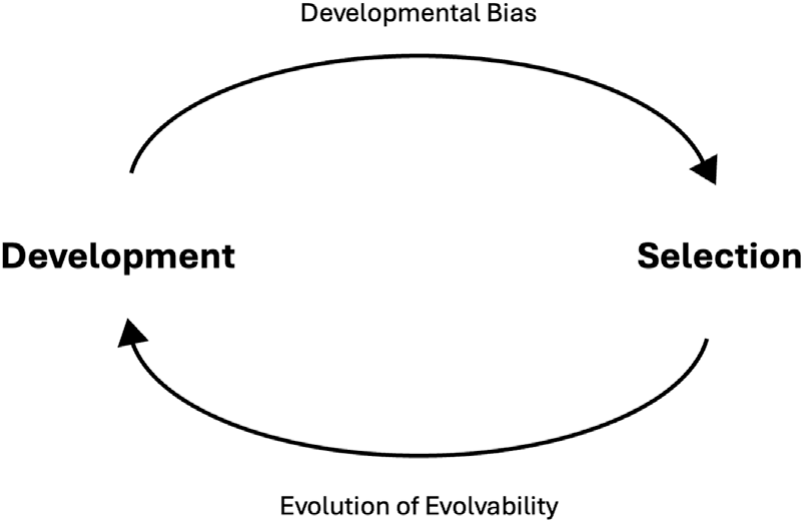
Reciprocity between development and selection drives evolution. Development influences selection by “biasing” the phenotypic variation that is made available (top arrow). When selection acts on phenotypic variation, it is also acting the developmental processes that generate said variation. Hence developmental biases can confer evolvability, and this evolvability is under selection and thus capable of evolving (bottom arrow). By acting on developmental processes and evolvability, selection influences the distribution of phenotypic variation in the next generation, thus what variation is exposed to selection.

We therefore argue that empirical and theoretical data point to the conclusion that developmental bias is both a cause and consequence of adaptive radiation and divergence. Rather than viewing development and selection as separate and/or conflicting processes, we recognize the inherent reciprocity present here that drives evolution (Figure 3). Development determines what variation is made available for selection, while selection acts both on variation and the developmental system that has produced it. Hence, future evolution will be driven by variation produced by a biased developmental system that is the product of selection. Thus, the role of development in evolution should be expanded from just being a driver of evolutionary change, to acknowledging that development, and its biases, are also the consequence of evolution. As Jones et al. (2004) say, “The response of a population to selection is a consequence of selection.”

## 4 Discussion

In this review we have described evolutionary patterns – parallel evolution, rapid diversification and phenotypic plasticity – that have for decades been attributed to random mutations and the guiding hand of selection. We therefore outline a set of questions that aim to demonstrate that developmental bias can explain the accelerated and exaggerated patterns of evolution that characterise adaptive radiations. While we have provided findings from several distinct fields that support these conclusions, the lack of explicit links between developmental bias and adaptive radiations leaves a number of key questions unanswered.

### 4.1 Macroevolutionary consequences of developmental bias

While developmental bias has been empirically demonstrated at the microevolutionary level (Braendle et al., 2010; McGlothlin et al., 2018; Rohner et al., 2022), its macroevolutionary consequences have only been tentatively suggested (e.g., Houle et al., 2017), as in this review. This represents a major gap in our understanding, as without knowledge of the stability of these biases we can only hypothesize about their role in evolutionary change. One way to bridge this gap would be to use gene-editing, RNAi, or mutagenic substances to induce mutations across a radiating lineage in the lab and observe how development is perturbed. If mutations, especially in different genes, bias developmental noise along axes corresponding to macroevolutionary patterns within said lineage, this would provide strong support that such biases are channeling long-term evolutionary change. For example, Rohner et al. (2022) used RNAi to disrupt distinct signalling pathways in the dung beetle *Onthophagus taurus* and observed that i) independent mutations produced similar phenotypic consequences and ii) these patterns aligned with phylogenetic trends observed across the *Onthophagus* lineage. Deploying and expanding this approach in different radiating lineages would provide a clearer picture of how biases influence evolution over longer timeframes.

### 4.2 Biased plasticity and adaptive divergence

Phenotypic plasticity undoubtedly can play a driving role in adaptive radiations, but whether plastic responses are a consequence of developmental bias is still unclear. Thus, demonstrating biased plastic responses will only strengthen links between bias and adaptive radiations. These links can be tested by exposing a radiating species to an environmental cue and observing if the developmental responses mirror macroevolutionary patterns. Such findings would provide support for the flexible stem model of plasticity-led evolution, as has been done in numerous lineages (McGlothlin et al., 2018; Parsons et al., 2016; Wund et al., 2008). Whether genetic and environmental perturbations lead the production of variation in similar phenotypic directions has seldom been investigated (but see Rohner et al., 2022), but would lend support to theories tying developmental system properties to macroevolutionary patterns. Furthermore, whether different environmental variables induce similar developmental responses has not, to our knowledge, yet been investigated. The threespine stickleback may be an excellent system in which to test this, as they show similar patterns of divergence in response to different environmental gradients (Roberts Kingman et al., 2021), with plasticity known to be central to such responses (Pilakouta et al., 2023; Skúlason et al., 2019; Wund et al., 2008). If the observed ecotypic divergence is indeed mediated through shared patterns of trait covariation, if and how these covariation structures respond to external cues would give further insight into mechanisms controlling divergence (Navon et al., 2021).

### 4.3 Bias as consequence and cause of adaptation

Our central thesis in this review has been that developmental bias can influence, and be influenced by, evolutionary history to shape patterns of adaptive divergence. While we have provided examples from the literature that support this claim of reciprocity, explicit testing of these hypotheses is required. Comparisons of radiating and non-radiating lineages should be further utilised to determine the role of development. If evolvability with respect to key evolutionary innovations could be demonstrated in radiating lineages, but not in non-radiating ones, this would suggest links between evolvability and evolutionary patterns. These fundamental questions can also be studied by using lineages that have evolved in parallel, or alternatively, lineages we might expect to have diverged in this way but have “failed” to do so. If parallel evolution can be linked to similarly biased developmental systems, or if non-parallel evolution can be attributed to a lack of evolvability, this would implicate a role for bias in facilitating and/or preventing parallel evolution and adaptive divergence. Conversely, comparing populations with known evolutionary histories may inform us of how past evolutionary shifts have influenced evolvability, and thus may influence future divergence. Experimental evolution, in which model organisms are exposed to an artificial selection regime, may allow links between selection and developmental variability to be solidified. Understanding how developmental factors can influence the predictability of evolution could then be utilised to predict how species respond to pressing ecological threats such as climate change (Campbell et al., 2017; Feiner et al., 2021), invasive species (Cordeschi et al., 2022), and increasing urbanisation (Thompson et al., 2022).

### 4.4 Bias and speciation

With the greatly accelerated speciation rates seen in adaptive radiations (Schluter, 2000), how this phenomenon could be influenced by developmental bias is unknown and seldom considered. If populations are diverging towards different ends of the same ecological axes (e.g., ecotype formation), then this initial process could be accelerated through developmental bias. This would especially be true if divergence is seeded by biased phenotypic plasticity, and/or utilises developmental axes used in the lineage’s evolutionary history (Parsons et al., 2020). However, if and how developmental biases drive reproductive isolation, perhaps through reduced hybrid fitness, is unknown. Understanding the role of biases in driving speciation is an essential step in understanding how such biases drive adaptive radiations.

### 4.5 Bias and selection

While selection and development have been largely considered as antagonistic forces, with the former permitting evolution and the latter providing limits (Maynard-Smith et al., 1985), we have drawn on evidence from learning theory to demonstrate that this dichotomy is unhelpful (Kouvaris et al., 2017; Pavlicev et al., 2011). Instead, theoretical models suggest that development is influenced by past selection (Pavlicev et al., 2011; Watson and Szathmáry, 2016), and that the phenotypic variation that selection is given is influenced by developmental parameters (Machado et al., 2023; Mongle et al., 2022). However, if and how developmental biases interact with selection, and how this can influence broader evolutionary patterns, has rarely been studied outside of computational systems (but see Walter et al., 2018). Thus, how malleable biases are in the face of selection, and how stable such biases are over evolutionary time, are important questions pertaining to the evolutionary consequences of developmental biases.

## 5 Conclusion

In this paper we have outlined a prominent role for developmental bias as a driver of many of the hallmark features of adaptive radiations. In the process, we have cited studies on many evolutionary topics, such as parallel evolution and phenotypic plasticity, that we argue are studying developmental bias, although this term may not be used directly. We may then suggest that a failure to invoke developmental explanations results not from a lack of empirical evidence, but from under-appreciating the role of development in adaptive evolution. By discussing developmental biases in the context of adaptive radiations, and their concomitantly exaggerated patterns of evolution, we hope to exemplify that biases do not merely constrain adaptive evolution but can also facilitate it. These astounding evolutionary events should also arm us with data and discoveries that can allow a proper understanding of development’s role in evolution.
